# CYFRA 21-1 level predicts survival in non-small-cell lung cancer patients receiving gefitinib as third-line therapy

**DOI:** 10.1038/sj.bjc.6602296

**Published:** 2004-12-14

**Authors:** F Barlési, C Tchouhadjian, C Doddoli, J-P Torre, P Astoul, J-P Kleisbauer

**Affiliations:** 1 Department of Thoracic Oncology, Faculty of Medicine – Assistance Publique Hôpitaux de Marseille, Fédération des Maladies Respiratoires, Hôpital Sainte-Marguerite, 270, Bd de Sainte-Marguerite, 13274 Marseille Cedex 09, France; 2 Department of Thoracic Surgery, Faculty of Medicine – Assistance Publique Hôpitaux de Marseille, Hôpital Sainte-Marguerite, 13274 Marseille Cedex 09, France; 3 Département d'Information Médicale, Assistance Publique Hôpitaux de Marseille, Hôpital de la Timone, 13385 Marseille Cedex 5, France

**Keywords:** non-small-cell lung cancer, CYFRA 21, gefitinib, ZD 1839, prognostic factors

## Abstract

Epidermal growth factor receptor (EGFR) tyrosine kinase inhibitors (TKI) as gefitinib emerged as an accepted treatment in second- or third-line setting in NSCLC. However, clinical surrogate markers of EGFR-TKI activity in NSCLC patients remain to be identified and we studied the prognostic value of CYFRA 21-1 in this setting. Serum samples from 53 patients with NSCLC receiving gefitinib after failure of at least a platinum-containing regimen were prospectively collected from January 2002 to December 2003. Multivariate analysis demonstrated an independent negative impact on survival for a level of CYFRA 21-1 higher than 3.5 ng ml^−1^ (HR=2.45, 95% CI 1.13–5.29; *P*=0.02). In conclusion, CYFRA 21-1 is a tool available to predict the survival of NSCLC patients receiving gefitinib as third-line therapy in an independent manner. In case of a CYFRA 21-1 level higher than 3.5 ng ml^−1^, treatment with gefitinib needs further evaluation giving its relative poor effect on survival.

The prognostic value of CYFRA 21-1 for non-small-cell lung cancer (NSCLC) patients was recently definitively demonstrated in a meta-analysis in 2063 patients (Pujol *et al*, 2004). In the same time, epidermal growth factor receptor (EGFR) tyrosine kinase inhibitors (TKI) as gefitinib emerged as an accepted treatment in second- or third-line setting in NSCLC (Pfister *et al*, 2004). However, clinical surrogate markers of EGFR-TKI activity in NSCLC patients after failure of platinum-containing regimen remain to be identified. So, we conducted a study of the prognostic value of CYFRA 21-1 in NSCLC patients receiving gefitinib after failure of platinum-containing regimen in an expanded access program.

## PATIENTS AND METHODS

Serum samples from patients with NSCLC receiving gefitinib after failure of at least a platinum-containing regimen were prospectively collected from January 2002 to December 2003. Histological subclassification was carried out according to the World Health Organization classification. Performance Status was estimated using the Eastern Cooperative Oncology Group (ECOG) scale. Clinical examination, chest, abdomen and brain-computed tomographic scan were carried out systematically.

Serum samples were obtained from each patient before gefitinib therapy. Fresh serum was collected and cooled after sampling, and then stored at −20°C until analysis. All assays were performed using commercial kit (ELSA CYFRA 21-1 CisBiointernational™) blind to clinical information. Serum level of CYFRA 21-1 was considered as elevated when it was superior or equal to 3.5 ng ml^−1^. Cutoff value of 3.5 ng ml^−1^ for CYFRA 21-1 was based on previously published results (Reinmuth *et al*, 2002). All patients consented to treatment with gefitinib.

Survival data were updated in June 2004. One patient was lost. Probability of survival was estimated using the Kaplan–Meier method. Differences between survival were tested by means of log-rank test. A multivariate regression analysis was carried out with Cox's regression using the forward maximum-likelihood method. All variables with a *P*-value less than 0.20 at the time of univariate analysis were entered into the model. A *P*-value less than 0.05 was considered as significant.

## RESULTS

In all, 53 patients were included in the study (Table 1). Two patients did not received gefitinib due to disease progression and were excluded from analysis. The median age of the 51 patients was 60 years (38–78 years). All the patients were current or former smokers. All the patients received a two-drugs platinum-containing regimen as first line of treatment. A total of 47 (92.1%) received a third-generation drug regimen (gemcitabine, docetaxel or paclitaxel) in both first- and second-line therapy. A skin toxicity of grade 1 and 2 occurred for 11 (21.6%) and two (3.9%) patients, respectively. Diarrhoea occurred for eight patients (15.7%). The median duration of treatment was 3.3 months. The disease control rate was 70.6%, with 11.7% of partial response and 58.9% of stable disease. At the time of analysis, 33 patients were deceased. The median survival time was 4 months (1 to 20+ months).

The principal results of univariate analysis are shown in Table 2. Multivariate analysis demonstrated an independent favourable impact on survival for occurrence of a skin reaction (HR=0.28, 95% CI 0.12–0.66; *P*=0.004) and an independent negative impact on survival for a level of CYFRA 21-1 higher than 3.5 ng ml^−1^ (HR=2.45, 95% CI 1.13–5.29; *P*=0.02).

## DISCUSSION

This study highlights the role of CYFRA 21-1 level in assessing prognosis of NSCLC patients receiving gefitinib as third-line therapy (HR for death is 2.45 for patients with CYFRA 21-1 level higher than 3.5 ng ml^−1^, *P*=0.02). Giving this result, CYFRA 21-1 could be used as a help in determining patients who beneficiate from treatment with gefitinib. This could be of a paramount importance giving the lack of clinical factors in this setting.

Whereas genetic mutations on EGFR gene receptor were identified in tumours of patients responding to gefitinib (Lynch *et al*, 2004; Paez *et al*, 2004), we also need more easily assessable and clinically available predictive factors for response to gefitinib for daily practice. Using commercial kit, CYFRA 21-1 is easily assessable and its role as prognostic marker alone (Pujol *et al*, 2004) or in combination (Barlési *et al*, 2004) has been demonstrated. Furthermore, the prognostic value of CYFRA 21-1 in the present study was higher than that of clinical (performance status, weight loss) or biological (leucocytes, liver enzymes) traditional prognostic markers. In addition, previous studies interested in clinical (gender, Karnofsky index) or biological (EGFR expression, HER2 expression) factors predictive for response or survival for patients treated with gefitinib reported contradictive or negative results, except for those with adenocarcinoma histological subtype and nonsmoking history, which predict a better survival (Miller *et al*, 2004). Likewise, skin rash has been suggested to predict response to gefitinib as we have shown in our multivariate analysis; however, available data do not definitively support this hypothesis (van Zandwijk, 2003).

In conclusion, CYFRA 21-1 is a tool available to predict survival of NSCLC patients receiving gefitinib as third-line therapy in an independent manner. So, CYFRA 21-1 should be integrated in the biological assessment of NSCLC patients prior to receiving gefitinib. In case of a CYFRA 21-1 level higher than 3.5 ng ml^−1^, treatment with gefitinib needs further evaluation giving its relative poor effect on survival.

## Figures and Tables

**Table 1:** Major clinical and biological factors of the study population

	***n* (%)**
Age <70/⩾70 years	38 (74.5)/13(25.5)
Gender women/men	16 (31.4)/35 (68.6)
Performance status 0–1/⩾2 (ECOG)	37 (72.5)/14 (27.5)
>5% weight loss	11 (21.6)
*Histology*	
Squamous cell carcinoma	15 (29.4)
Adenocarcinoma	24 (47.1)
Large-cell carcinoma	12 (23.5)
	
Stage^a^ IIIB/V	13 (25.5)/38 (74.5)
*Chemotherapy regimens before gefitinib*	
2	30 (58.8)
3	15 (29.4)
⩾4	6 (11.8)
	
Leucocytes (G l^−1^) <10.5/⩾10.5	42 (82.4)/9 (17.6)
LDH (IU l^−1^) <500/⩾500	37 (72.5)/14 (27.5)
Calcium level (mmol l^−1^) <2.75/⩾2.75	48 (94.1)/3 (5.9)
Hepatic enzymes level (UI l^−1^) <2 × ULN/⩾2 × ULN	46 (90.2)/5 (9.8)
CYFRA 21-1 level (ng ml^−1^) <3.5/⩾3.5	38 (74.5)/13 (25.5)

a UICC classification; LDH=lactate dehydrogenase; ULN=upper limit of the normal range; ECOG=Eastern Cooperative Oncology Group.

**Table 2:** Results of univariate analysis (only factors with *P*<0.02 were reported)

	**Median survival (months)**	***P*-value**
PS <2/⩾2	7/2	0.0541
Weight loss (yes/no)	2/6	0.0568
Hepatic enzymes level <2 × ULN/⩾2 × ULN	4/2	0.1165
CYFRA 21-1 level (ng ml^−1^) <3.5/⩾3.5	7/2	0.0208
Leucocytes (G l^−1^) <10.5/⩾10.5	4/2	0.1723
Skin reaction (yes/no)	15/3	0.0056

PS=performance status (ECOG); ULN=upper limit of the normal range.
